# The p38 MAPK Inhibitor SB203580 Abrogates Tumor Necrosis Factor-Induced Proliferative Expansion of Mouse CD4^+^Foxp3^+^ Regulatory T Cells

**DOI:** 10.3389/fimmu.2018.01556

**Published:** 2018-07-09

**Authors:** Tianzhen He, Shuoyang Liu, Shaokui Chen, Jingyi Ye, Xueqiang Wu, Zhaoxiang Bian, Xin Chen

**Affiliations:** ^1^State Key Laboratory of Quality Research in Chinese Medicine, Institute of Chinese Medical Sciences, University of Macau, Macau SAR, China; ^2^Department of Oncology, Beijing Aerospace General Hospital, Beijing, China; ^3^School of Chinese Medicine, Hong Kong Baptist University, Kowloon, Hong Kong SAR, China

**Keywords:** tumor necrosis factor, TNF receptor type II, p38 MAPK, CD4^+^Foxp3^+^ regulatory T cells, proliferation

## Abstract

There is now compelling evidence that tumor necrosis factor (TNF) preferentially activates and expands CD4^+^Foxp3^+^ regulatory T cells (Tregs) through TNF receptor type II (TNFR2). However, it remains unclear which signaling transduction pathway(s) of TNFR2 is required for the stimulation of Tregs. Previously, it was shown that the interaction of TNF–TNFR2 resulted in the activation of a number of signaling pathways, including p38 MAPK, NF-κB, in T cells. We thus examined the role of p38 MAPK and NF-κB in TNF-mediated activation of Tregs, by using specific small molecule inhibitors. The results show that treatment with specific p38 MAPK inhibitor SB203580, rather than NF-κB inhibitors (Sulfasalazine and Bay 11-7082), abrogated TNF-induced expansion of Tregs *in vitro*. Furthermore, upregulation of TNFR2 and Foxp3 expression in Tregs by TNF was also markedly inhibited by SB203580. The proliferative expansion and the upregulation of TNFR2 expression on Tregs in LPS-treated mice were mediated by TNF–TNFR2 interaction, as shown by our previous study. The expansion of Tregs in LPS-treated mice were also markedly inhibited by *in vivo* treatment with SB203580. Taken together, our data clearly indicate that the activation of p38 MAPK is attributable to TNF/TNFR2-mediated activation and proliferative expansion of Tregs. Our results also suggest that targeting of p38 MAPK by pharmacological agent may represent a novel strategy to up- or downregulation of Treg activity for therapeutic purposes.

## Introduction

CD4^+^Foxp3^+^ regulatory T cells (Tregs) are crucial for the maintenance of immune homeostasis and for the prevention of autoimmune responses (1). They also play a major role in immune evasion of cancer by dampening immune responses against tumor (2). Targeting Tregs has become a strategy in the treatment of major human diseases, such as cancer, allergic and autoimmune diseases, transplantation rejection, and GVHD (3). A thorough understanding of biological pathways that regulate Treg function is a prerequisite for the up- or downregulation of Treg activity for therapeutic purposes.

We (Xin Chen and Joost J. Oppenheim) for the first time report that tumor necrosis factor-alpha (TNF) can activate Tregs through TNF receptor type II (TNFR2), one of TNF receptors, which is preferentially expressed by Tregs (4). Furthermore, we found that expression of TNFR2 identifies the maximally potent suppressive human and mouse Treg subsets (5, 6). In contrast, Tregs without TNFR2 expression only had minimal or no suppressive activity (5, 7, 8). Moreover, TNF–TNFR2 signaling is important for the phenotypical stability of Tregs, including Foxp3 expression (4, 8, 9). The notion that TNF–TNFR2 signaling plays a decisive role in the activation, expansion, and phenotypical stability of Tregs is now supported by compelling evidences from other groups (10–21). Nevertheless, which signaling transduction pathway(s) of TNFR2 is required for Treg-stimulatory effect of TNF remains unknown.

The biological functions of TNF are transduced by two receptors, TNFR1 (p55) and TNFR2 (p75) (22). In contrast to the ubiquitous expression of TNF receptor type I (TNFR1), TNFR2 is mainly expressed by lymphocytes (23). Signal transduction by TNFR1 has been intensively investigated and well defined, while the TNFR2 signaling pathway is less well understood (24). So far, three signaling pathways of TNFR2 in T lymphocytes have been documented, including IKK/NF-κB, MAPK (Erk1/2, p38, JNK), and PI3K/Akt pathways (25, 26). Previously, p38 MAPK signaling pathway has been shown to play a key role in the immunosuppressive function of induced Tregs (iTregs) in both *in vitro* and *in vivo* studies (27–29). It was also reported that inhibition of p38 MAPK signaling was able to reduce immunosuppression of iTregs on Teffs, and consequently enhanced antitumor immune responses (29, 30). It has been shown that TNF stimulation resulted in the activation of p38 MAPK, in addition to the activation of NF-κB, in Tregs (31, 32). Thus, we hypothesized that p38 MAPK signaling pathway may be also attributable to the activation and proliferation of Foxp3^+^ naturally occurring Tregs (nTregs) by TNF–TNFR2 interaction.

In this study, we investigated the effect of SB203580, a p38 MAPK-specific inhibitor, on the expansion of Tregs induced by the interaction of TNF–TNFR2 in both *in vitro* and *in vivo* experimental settings. The results showed that SB203580 potently inhibited TNF-induced proliferative expansion of Tregs. Furthermore, other stimulatory effects of TNF on Tregs, such as upregulation of TNFR2 and Foxp3 expression were also abrogated by SB203580. Therefore, p38 MAPK represents a major component of signaling pathway of TNFR2 in the activation of Tregs.

## Results

### SB203580 Inhibits TNF-Induced Proliferation of Tregs *In Vitro*

**Figure 1:**
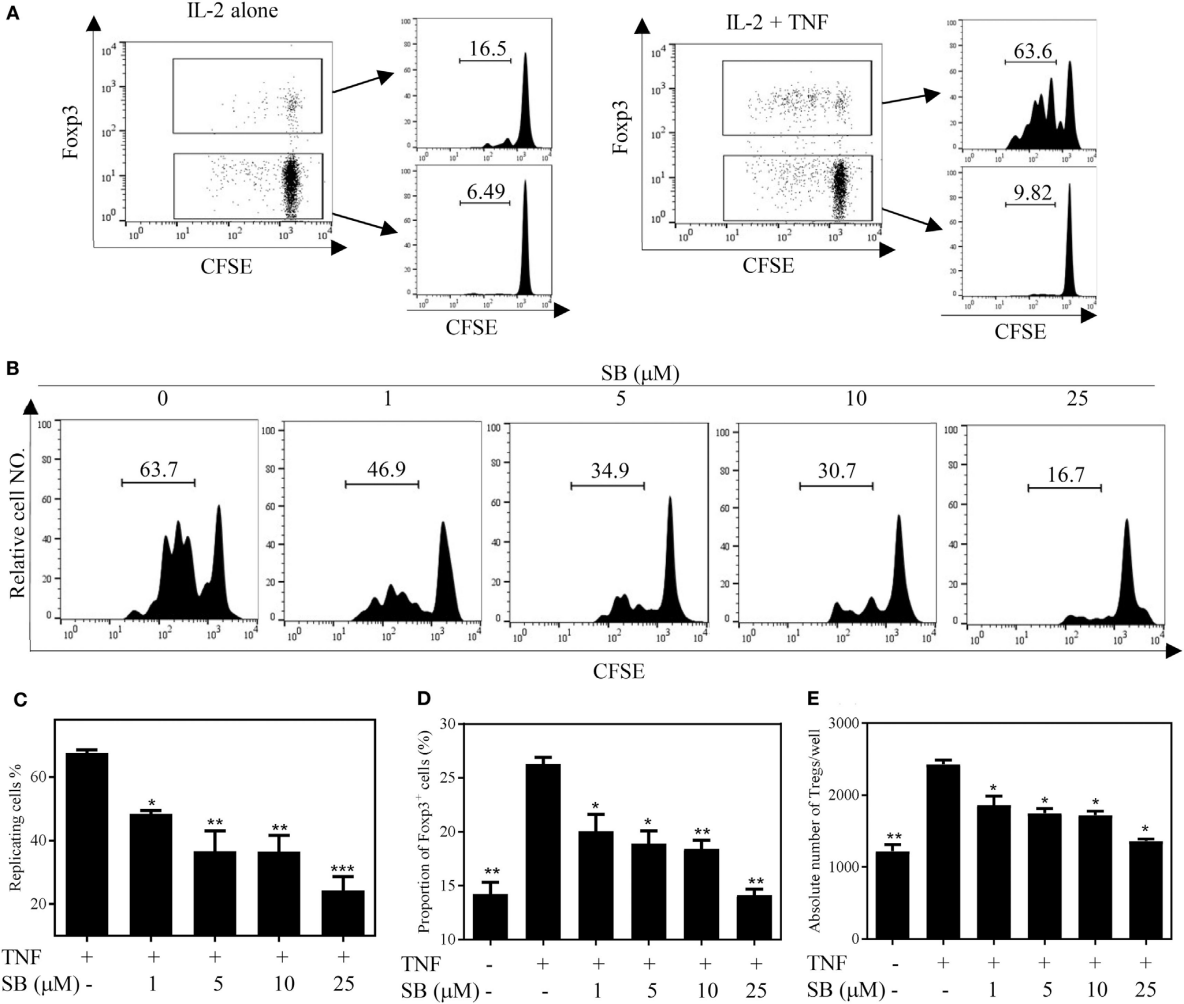
SB203580 (SB) inhibits tumor necrosis factor (TNF)-mediated expansion of regulatory T cells (Tregs) *in vitro*. CD4^+^ T cells were purified from LNs and spleen of normal C57BL/6J mice by MACS. The cells were labeled with CFSE and cultured in the presence of IL-2 (10 ng/mL), or IL-2 + TNF (10 ng/mL, each), with medium alone or with different concentrations of SB203580 (SB, 1, 5, 10, and 25 µM). After 72 h, the proliferation of Tregs and the proportion of Foxp3^+^ cells were analyzed by FACS, based on CFSE expression and Foxp3 expression. The absolute number of Foxp3-expressing Tregs was calculated. **(A)** In the presence of IL-2, TNF preferentially stimulated the proliferation of Tregs. **(B,C)** SB203580 blocked TNF-mediated proliferation of Tregs. Analysis was gated on Foxp3^+^ Tregs. **(D)** SB203580 decreased the proportion of Foxp3^+^ Tregs in the cultured CD4^+^ T cells. **(E)** SB203580 reduced the absolute number of Tregs in the cultured CD4^+^ T cells. **(A,B)** Show the typical FACS plots. The number in the histogram indicates the proportion of gated cells (%). **(C,D)** Show the summary of results (*N* = 3, means ± SEM). By comparison with “TNF + IL-2” group, *p < 0.05, **p < 0.01, ***p < 0.001. Data shown are representatives of at least three separate experiments with similar results.

**Figure 2:**
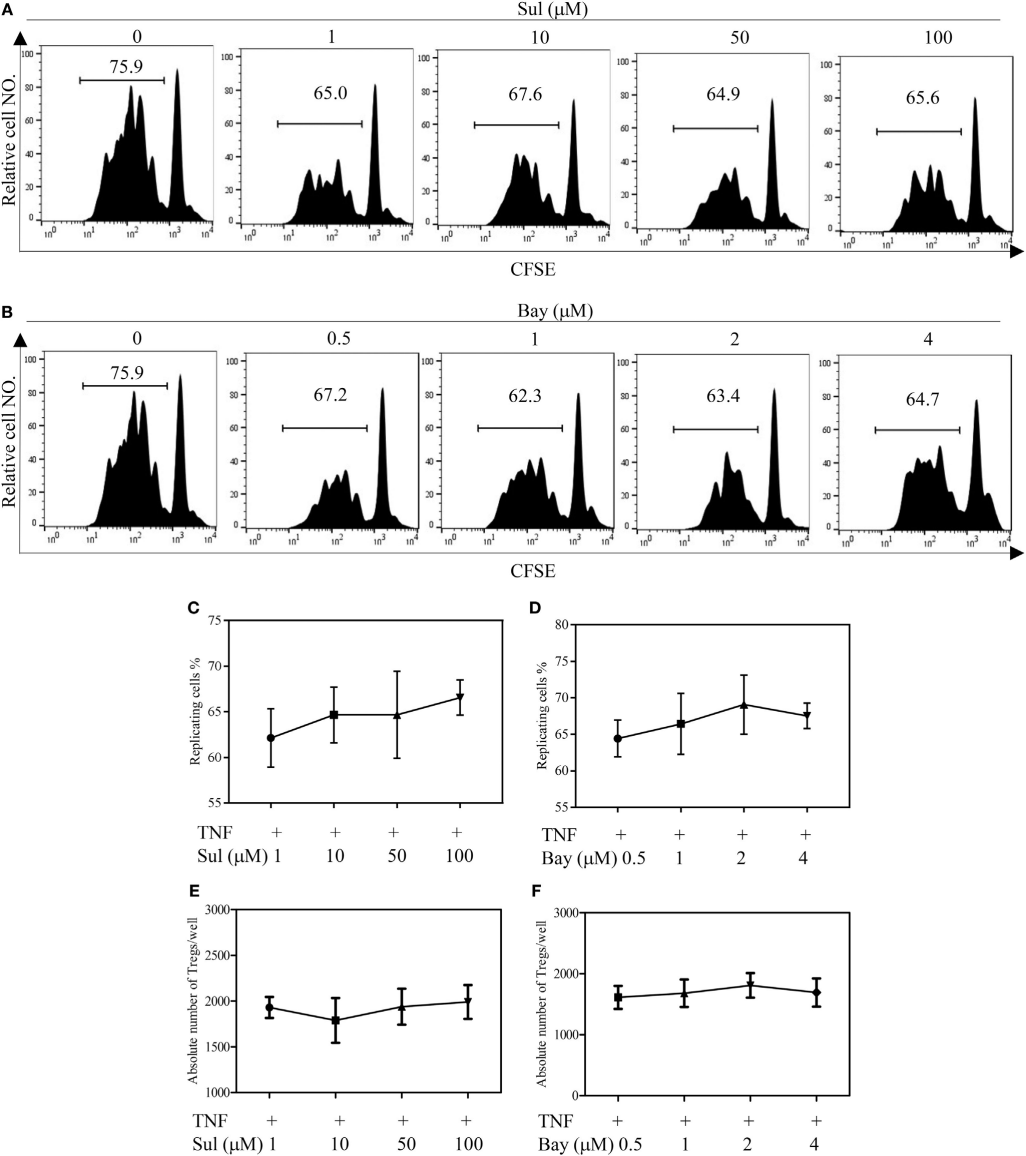
Effect of NF-κB inhibitors on tumor necrosis factor (TNF)-mediated proliferative expansion of regulatory T cells (Tregs). CD4^+^ cells were purified from LNs and spleen of normal C57BL/6J mice by MACS. The cells were labeled with CFSE and cultured in the presence of IL-2 (10 ng/mL), or IL-2 + TNF (10 ng/mL, each), with medium alone or with different concentrations of Sulfasalazine (Sul, 1, 10, 50, 100 µM) or Bay 11-7082 (Bay, 0.5, 1, 2, and 4 µM). After 72 h, the proliferation of Tregs and the absolute number of Foxp3^+^ cells were analyzed by FACS, based on CFSE expression and Foxp3 expression. **(A,B)** Typical FACS analysis of Treg proliferation, as shown by dilution of CFSE expression (gating on Foxp3^+^ cells). The number in the histogram indicates the proportion of gated cells, e.g., replicating cells (%). **(C,D)** The summary of proportion of replicating Tregs. **(E,F)** The absolute number of Treg cells per well. Data shown in **(C–F)** are representatives of at least three separate experiments with similar results (*N* = 3, means ± SEM).

We firstly examined the *in vitro* effect of p38 MAPK-specific inhibitor SB203580 (33) on the expansive proliferation of Tregs induced by TNF. To this end, CD4^+^ T cells were purified by MACS from spleen and LNs of normal mice. The cells were cultured with IL-2 to maintain their survival (34). Consistent with our previous report (4, 17), addition of TNF preferentially stimulated the proliferation of Tregs, resulting in proliferation of greater than 60% of Tregs (Figure 1A). Consequently, the absolute number of Tregs in the cultured CD4^+^ T cells was increased twofold by TNF stimulation (Figure 1E). As shown in Figures 1B–C, in a concentration range of 1–25 µM, SB203580 inhibited the TNF-induced proliferation of Tregs in a dose-dependent manner, with a percent inhibition of 32.0–73.2% (*p* < 0.05–0.001). The proportion of Foxp3^+^ Tregs in the cultured CD4^+^ T cells was also markedly reduced by SB203580 treatment, with a percent inhibition of 24.9–47.05% (Figure 1D, *p* < 0.05–0.01). Furthermore, the absolute number of Tregs in each well was markedly reduced (Figure 1E, *p* < 0.05). In contrast, treatment with two NF-κB inhibitors [Sulfasalazine (35) and Bay 11-7082 (36)] failed to inhibit TNF-induced proliferative expansion of Tregs in the cultured CD4^+^ T cells (Figures 2A–F). These results suggest that the activation of p38 MAPK, rather than the activation of NF-κB, is required for the proliferative expansion of Tregs triggered by TNFR2 signaling. Treatment with SB203580 in the concentration range used in our *in vitro* study did not induce cell death (Figure S1 in Supplementary Material). Furthermore, SB203580 treatment did not reduce the number of Tregs in CD4 T cells cultured with IL-2 alone (Figure S2 in Supplementary Material). These data exclude the possibility that the inhibitory effect of SB203580 was based on the cytotoxic effect.

### SB203580 Downregulates TNFR2 Surface Expression on TNF-Stimulated Tregs

**Figure 3:**
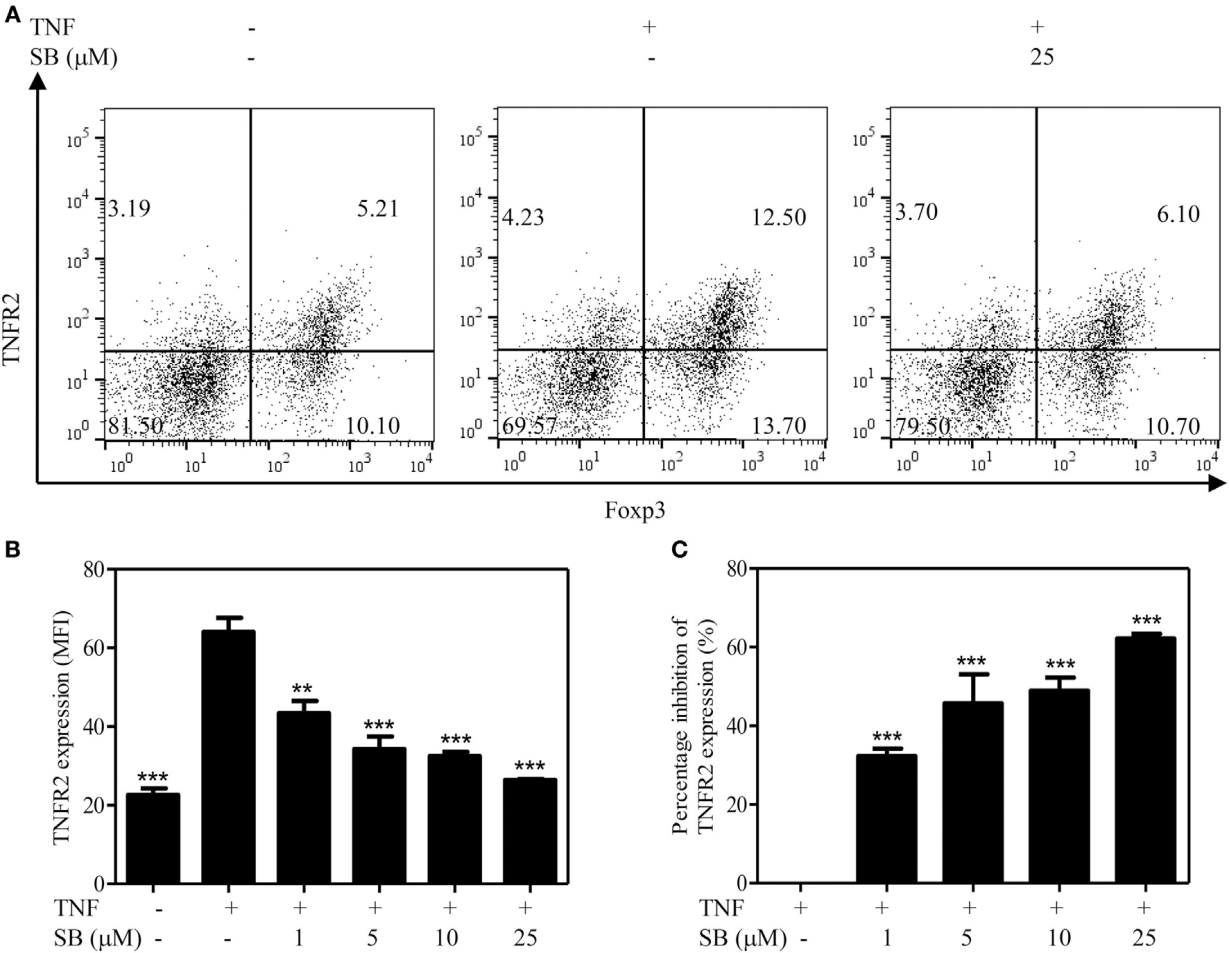
Upregulation of TNFR2 expression on regulatory T cells (Tregs) induced by tumor necrosis factor (TNF) is abrogated by SB203580. MACS-purified CD4^+^ T cells were cultured in the presence of IL-2 (10 ng/mL), or IL-2 + TNF (10 ng/mL, each), with medium alone or with SB203580 (1–25 µM). The cells were cultured for 72 h. The surface expression of TNFR2 and intracellular expression of Foxp3 were analyzed with FACS. **(A)** Typical FACS dot plot of TNFR2 and Foxp3 expression. Data shown are representatives of at least three separate experiments with similar results. Number in the FACS plot shows the proportion of cells in the respective quadrants. **(B)** Summary of mean fluorescence intensity (MFI) of TNFR2 expression on Tregs (by gating on Foxp3^+^ cells. *N* = 3, means ± SEM). **(C)** Percent inhibition of TNFR2 expression on Foxp3^+^ Tregs (*N* = 3, means ± SEM). The formula used to calculate percent inhibition is: (A − B)/A × 100%, A is MFI of TNFR2 expression treated with TNF/IL-2, B is MFI of TNFR2 expression treated with SB203580 (1–25 µM) + TNF/IL-2. By comparison with “TNF + IL-2” group, **p < 0.01, ***p < 0.001. Data shown are representatives of at least three separate experiments with similar results.

The surface expression levels of TNFR2 are correlated with immunosuppressive function of Tregs (5, 6). Previously, we showed that treatment with TNF preferentially upregulates TNFR2 expression on Tregs (37). To determine if p38 MAPK pathway plays a role in the upregulation of TNFR2 expression on Tregs, MACS-purified CD4^+^ T cells were cultured with IL-2, with or without TNF. The cells were treated with SB203580 (1–25 µM). As shown in Figure 3A, the treatment with TNF upregulated TNFR2 expression on Tregs by >2-folds, as compared with IL-2 cultured alone. TNF-induced upregulation of TNFR2 expression was inhibited by SB203580 in a dose-dependent manner (Figures 3A,B, *p* < 0.01–0.001), with a percent inhibition of 32.3–62.6% (Figure 3C, *p* < 0.01–0.001). Thus, inhibition of p38 MAPK with SB203580 can inhibit surface expression of TNFR2 on TNF-treated Tregs.

### SB203580 Abrogates TNF-Induced Upregulation of Foxp3 Expression in Tregs

**Figure 4:**
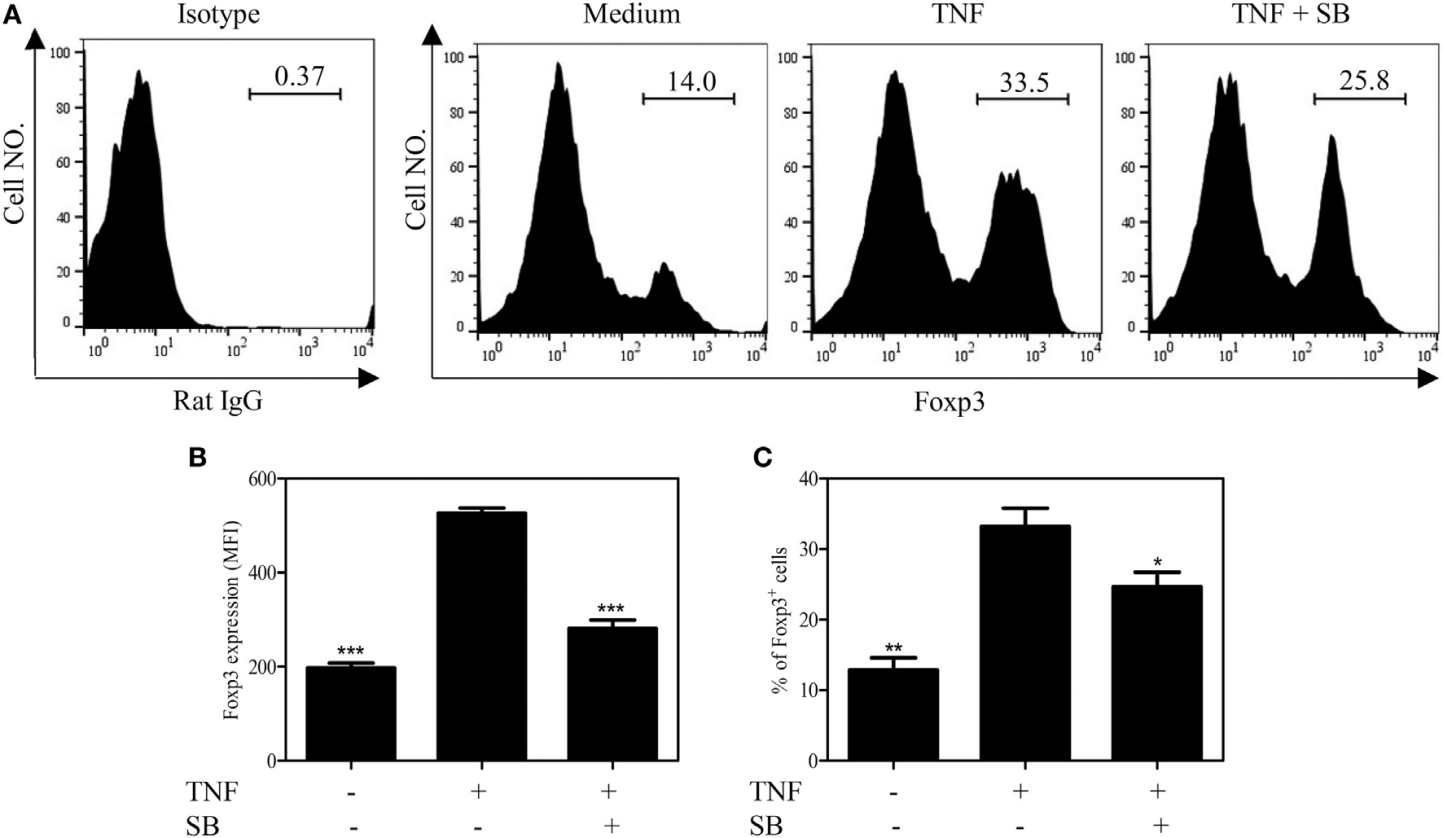
SB203580 inhibits Foxp3 expression in tumor necrosis factor (TNF)-treated regulatory T cells. FACS-sorted CD4^+^CD25^+^ T cells were stimulated with plate-bound anti-CD3 and soluble anti-CD28 Abs, in the presence or absence of TNF (10 ng/mL), with or without 25 µM SB203580 for 3 days. Foxp3 expression and ratio of Foxp3^+^ cells were analyzed by FACS. **(A)** Typical histograms of Foxp3 expression. Number in the histogram indicates the proportion of gated cells. **(B)** Summary of Foxp3 expression (MFI. *N* = 3, means ± SEM). **(C)** Summary of proportion of Foxp3-expressing cells (*N* = 3, means ± SEM). By comparison with TNF group (without SB203580), *p < 0.05, **p < 0.01, ***p < 0.001. Data shown are representatives of at least three separate experiments with similar results.

TNF–TNFR2 interaction is also crucial for the phenotype stability of Tregs, in term of Foxp3 expression, in both *in vitro* and *in vivo* settings (8). We thus examined the effect of SB203580 on Foxp3 expression by TCR-stimulated Tregs. To this end, mouse CD4^+^CD25^+^ T cells were flow-sorted and stimulated with plate-bound anti-CD3 Ab and soluble anti-CD28 Ab for 3 days, a known condition, which can downregulate Foxp3 expression (8). Treatment with the exogenous TNF could partially maintain Foxp3 expression (Figures 4A–C), consistent with our previous report (8). The levels of Foxp3 expression on per cell basis (MFI) and the proportion of Foxp3-expessing cells were increased by twofold after TNF treatment. These effects of TNF were largely abrogated by the treatment of SB203580 (Figures 4A–C). It is worth noting that SB203580, in the absence of TNF, did not downregulate Foxp3 expression in Tregs (Figure S2 in Supplementary Material).

### SB203580 Inhibits *In Vivo* Expansion of Tregs in LPS-Treated Mice

**Figure 5:**
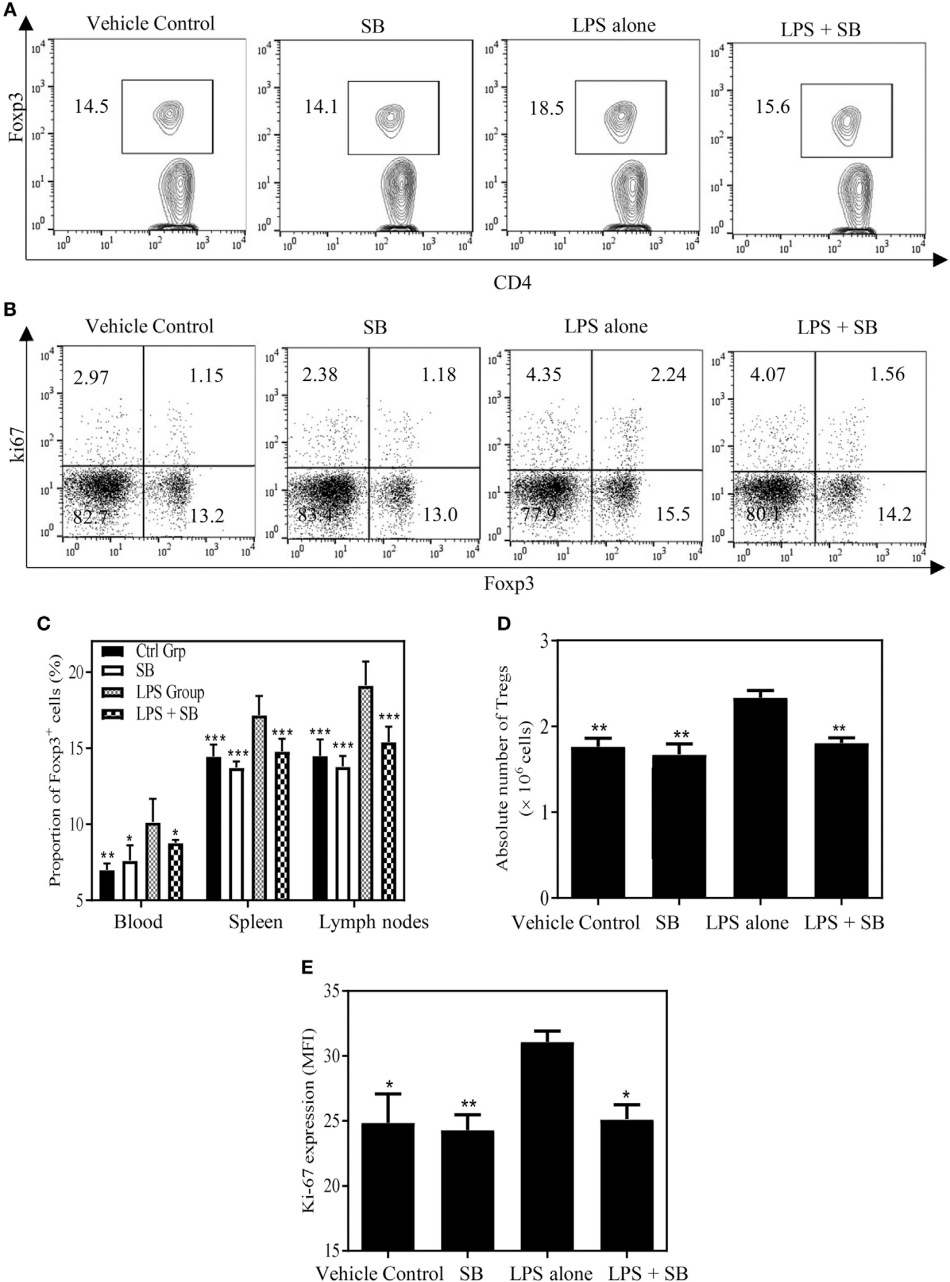
SB203580 inhibits expansion of regulatory T cells (Tregs) in LPS-treated mice. C57BL/6J mice were injected with 200 µg of LPS (i.p.) or PBS, and treated with or without SB203580 (25 mg/kg/day, i.p.) immediately after LPS challenge. All mice were sacrificed 24 h after LPS treatment. Blood, spleen, and lymph nodes were harvested. The proportion of Foxp3^+^ Tregs in CD4^+^ T cells and expression of Ki-67 by Tregs were analyzed by FACS, gating on Foxp3^+^ cells. The absolute number of Tregs was calculated. **(A)** Expression of Foxp3 by CD4^+^ T cells. Number shows the proportion of gated cells. **(B)** Expression of Ki-67 by Foxp3^−^ and Foxp3^+^ cells. Number shows the proportion of positive cells in the respective quadrants. **(A,B)** Typical FACS plots were shown. **(C)** Summary of proportion of Tregs in CD4^+^ T cells in the peripheral blood, spleen and LNs. **(D)** Summary of absolute number of Tregs in the spleen. **(E)** Ki-67 expression (MFI) by Foxp3^+^ Tregs. Data [means ± SEM) in **(C)** were pooled from three separate experiments (spleen and lymph nodes: *N* = 9, peripheral blood: *N* = 6), and in **(D,E)** (*N* = 3) were representatives of at least three separate experiments with similar results. By comparison with LPS alone group, *p < 0.05, **p < 0.01, ***p < 0.001.

**Figure 6:**
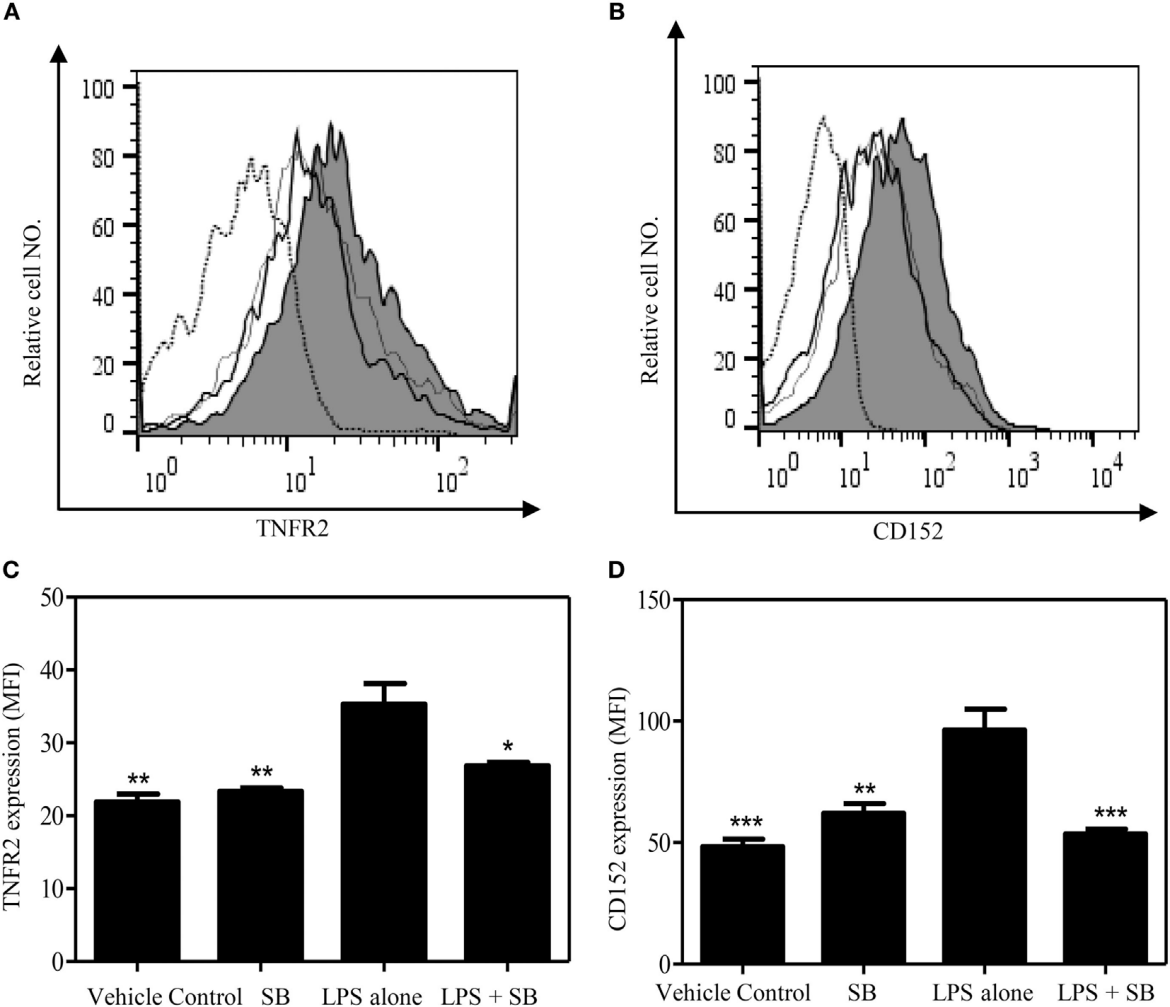
SB203580 inhibits the upregulation of TNFR2 expression and CD152 expression on regulatory T cells (Tregs) in LPS-treated mice. C57BL/6J mice were injected with 200 µg of LPS (i.p.) or PBS, and treated with or without SB203580 (25 mg/kg/day, i.p.). Mouse spleen were harvested at 24 h after injection for the FACS analysis of CD152 and TNFR2 expression, gating on Foxp3^+^ cells. **(A,B)** Typical FACS histograms were shown. Black solid line: vehicle control; gray-filled histogram: LPS treatment; hair line: LPS + SB203580; Dot histogram: isotype control. Summary TNFR2 expression [MFI. **(C)**] and CD152 expression [MFI, **(D)**] by Foxp3^+^ Tregs (*N* = 3, means ± SEM). Data shown are representatives of at least three separate experiments with similar results. By comparison with LPS alone group, *p < 0.05, **p < 0.01, ***p < 0.001.

Previously, we showed that TNF–TNFR2 interaction is responsible for LPS-induced proliferation of Tregs in mice (37). More recently, we observed that LPS treatment was able to markedly upregulate the expression of transmembrane TNF on dendritic cells (DCs), and such DCs potently stimulated the proliferation of Tregs (data not shown). Therefore, LPS-treated mice were used to examine if SB203580 had the *in vivo* activity to inhibit TNF-induced expansion of Tregs. As shown in Figures 5A,C, the proportion of Foxp3^+^ cells in splenic CD4^+^ T cells was increased from 14.6% in control mice to 18.6% in mice 24 h after LPS treatment (*p* < 0.01). Similarly, the proportion of Foxp3^+^ cells in CD4 T cells present in peripheral blood and lymph nodes following intraperitoneal LPS injection was also increased compared with control mice (Figure 5C). The expressions of Ki-67, an indicator of replicating cells, and TNFR2 were markedly increased in the splenic Tregs (Figures 5B,E and 6A,C. *p* < 0.01–0.05), which is consistent with our previous report (7). Since the proportion of Tregs were increased in all observed tissues, which was accompanied by the upregulation of Ki-67, we concluded that the increased number of Tregs in LPS-treated mice was resulted from the proliferative expansion through the interaction of TNF–TNFR2, rather than resulted from the redistribution or alteration of trafficking pattern of Tregs (37). LPS treatment also increased the absolute number of Tregs in spleen by ~1.5-fold (Figure 5D, *p* < 0.01). Treatment with single dose of SB203580 (25 mg/kg/day, i.p.) immediately after LPS treatment completely inhibited LPS-induced expansion of Tregs (Figure 5A). Moreover, LPS-induced upregulation of Ki-67 and TNFR2 expression on Tregs was also completely abrogated by the treatment of SB203580 (Figures 5B,E and 6A,C). The inhibitory effect of SB203580 on the proliferative expansion of Tregs, as indicated by the proportion of Foxp3^+^ Tregs and their Ki-67 expression, in LPS-treated mice could last for at least 72 h (Figure S3 in Supplementary Material). CD152 (CTLA4) is a characteristic marker and an effector molecule of Tregs. Expression of CD152 in Tregs was upregulated by LPS-treatment (Figures 6B,D, *p* < 0.001), and the elevation of CD152 expression in LPS-treated mice was completely abrogated by SB203580 treatment (Figures 6B,D). Therefore, SB203580 has both *in vitro* and *in vivo* activity in the inhibition of TNFR2-mediated activation and expansion of Tregs.

## Discussion

The p38 MAPK signaling pathway is known to play a key role in mediating the responses of mammalian cells to LPS stimulation (38), including production of TNF by LPS-treated macrophages (39). The activation of p38 MAPK contributes to the pathogenesis of autoimmune diseases, such as rheumatoid arthritis (RA) and inflammatory bowel disease; however, the results from clinical trials failed to show the therapeutic effect of p38 MAPK inhibitors on these inflammatory diseases (40). The p38 MAPK has a multifaceted role in CD4^+^ T cells (41), including the activation, cytokine expression, the responses to TCR/co-stimulation, and effector function of Th1 and Th2 cells (42). It was shown that inhibition of p38 MAPK with SB203580 induced immune tolerance in (NZB × NZW)F1 lupus-prone mice, which was purportedly attributable to the increased Treg activity (43). However, more evidence indicates that inactivation or inhibition of p38 MAPK dampens the suppressive function of induced Tregs (iTregs). For example, the number of Tregs was increased in mice with T cells deficient in p38α and p38β (44). Inhibition of p38 MAPK with SB203580 significantly abrogated chronic stress-induced differentiation of Foxp3^+^ iTregs (45). Furthermore, treatment with SB203580 inhibits the induction and function of human and mouse iTregs (27, 46, 47) and mouse IL-10-producing CD25^−^ suppressive CD4 T cells (29). To date, the effect of inhibition of p38 MAPK with SB203580 on naturally occurring Tregs (nTregs), especially in an *in vivo* experimental setting, remains unknown.

It has been shown that TNF–TNFR2 interaction was able to activate p38 MAPK pathway in T cells through activation of Syk protein tyrosine kinase (48). Nagar/Goldstein and colleagues examined TNF-induced gene transcription in flow-sorted human Tregs (31). GCBI analysis of GSE18893 file uploaded by Nagar/Goldstein and colleagues (https://www.ncbi.nlm.nih.gov/geo/query/acc.cgi?acc=GSE18893) indicated that both p38 MAPK pathway and NF-κB pathway in Tregs were markedly activated after TNF stimulation (Tables S1 and S2 in Supplementary Material). Recent evidence also showed that TNFR2-specific TNF-variant scTNF(143N/145R) treatment markedly activated p38 MAPK and NF-κB in purified human Tregs (32). In our study, small molecule inhibitors of p38 MAPK and NF-κB pathways, namely SB203580, Sulfasalazine, and Bay 11-7082, were employed to determine which TNFR2 signaling pathway is required for Treg expansion induced by TNF–TNFR2 interaction. Previously, SB203580 was well characterized as a specific p38 MAPK inhibitor (33), and Sulfasalazine was a specific inhibitor of NF-κB activation (35), while Bay 11-7082 was a direct inhibitor of IKK and thus inhibits the signal-induced nuclear translocation of NF-κB (36). These three compounds have been frequently used by investigators to study the effect of inhibition of p38 MAPK and NF-κB in T cells, including Tregs (27, 46, 47). We confirmed that p38 MAPK and canonical NF-κB pathways in Treg cells were activated by TNF stimulation. Furthermore, such upregulation of p38 MAPK and NF-κB activity could be potently inhibited by SB203580, Sulfasalazine, and Bay 11-7082, respectively (Figure S4 in Supplementary Material). Our study clearly shows that p38 MAPK-specific inhibitor SB203580, but not sulfasalazine nor Bay 11-7082, potently inhibited TNF-induced expansion, expression of TNFR2 and Foxp3 on Tregs in both *in vitro* and *in vivo* experiments. Our results thus provide clear evidence that p38 MAPK may represent an important component of TNFR2 signaling pathway in the activation and expansion of Tregs induced by TNF.

In our *in vitro* studies, IL-2 was used to maintain the survival of cultured T cells. Previously, we showed that in this *in vitro* culture system, TNF-induced proliferation of Tregs was independent of IL-2 (37). This conclusion was further substantiated by the studies from other groups (12, 49). Thus, inhibition of Treg proliferation by SB203580 is mainly achieved by blockade of p38 MAPK activity triggered by TNF–TNFR2 signaling. This idea is supported by the observation that SB203580 did not reduce the number of Tregs in CD4 T cells cultured with IL-2 alone (Figure S2 in Supplementary Material). Nevertheless, IL-2 and TCR/CD28 co-stimulation can also induce the activation of p38 MAPK pathway (50, 51) and can also stimulate the activation and expansion of Tregs (52, 53). Such effect of IL-2 and TCR/CD28 may also contribute to *in vivo* expansion of Tregs in the inflammatory condition, such as in mice treated with LPS. If this is the case, targeting of p38 MAPK may be able to block Tregs expansion induced by multiple signaling pathways.

Elimination of Treg activity, by either reducing their number or downregulating their immunosuppressive function, has become a strategy to enhance the efficacy of cancer therapy (54). Since TNFR2 signaling plays a crucial role in the activation and expansion of Tregs, the major component of TNFR2 signaling pathway responsible for Treg-stimulatory effect may be harnessed to modulate Treg activity. Recent study indicates that TNFR2 is an emerging target of cancer immunotherapy (15, 55). As suggested by our study, inhibition of p38 MAPK may enhance the efficacy of tumor immunotherapy by eliminating Treg activity. Interestingly, it was shown that inhibition of p38 MAPK with SB203580 markedly enhances DC’s capacity to activate Teffs and overcome Treg-mediated suppression, and consequently promote antitumor immune response (30, 56, 57). Thus, p38 MAPK inhibitors may be useful as an immune adjuvant to enhance the efficacy of tumor immunotherapy by simultaneously acting on both Tregs and DCs.

The rationale of development of p38 MAPK inhibitor as therapeutic agent is largely based on the idea that inhibition of p38 MAPK would inhibit the production of TNF (39), since anti-TNF biologics have been shown great success in the treatment of autoimmune inflammatory diseases (58). Although preclinical studies suggest that p38 MAPK inhibitors had therapeutic potential in the treatment of inflammatory diseases in animal model, such as collagen-induced arthritis (59) and experimental allergic encephalomyelitis (60); however, the subsequent clinical trials have generally failed (40). Moreover, treatment with p38 MAPK inhibitors has the potential to induce additional inflammatory responses in RA patients (61). One possibility raised by our studies is that attenuation of Treg activity through interruption of TNF–TNFR2 interaction might be related to the failure of clinical trials designed to examine the effect of p38 MAPK inhibitors in the treatment of chronic inflammatory diseases.

Taken together, our data clearly show that p38 MAPK inhibitor SB203580 has the capacity to abrogate TNF-induced proliferative expansion, expression of TNFR2 and Foxp3 on Tregs. The results suggest that p38 MAPK may represent a key component of TNFR2 signaling pathway, which is required for the activation and expansion of Tregs. Thus, p38 MAPK pathway may be a therapeutic target to enhance the efficacy of cancer immunotherapy by eliminating Treg activity and other immunosuppressive mechanisms, and this possibility should be addressed in the future study.

## Materials and Methods

### Mice and Reagents

Female wildtype (WT) C57BL/6J (8–12 weeks old) were provided by the Animal Facility of University of Macau. The animal study protocol was approved by Animal Research Ethics Committee of University of Macau. Antibodies purchased from BD Pharmingen (San Diego, CA, USA) consisted of PerCP-Cy5.5 anti-mouse CD3 (145-2C11), PE anti-mouse CD4 (GK1.5), PE anti-mouse CD120b/TNFR2 (TR75-89), PerCP-Cy5.5 anti-mouse CD25 (PC61), PE anti-mouse CD152 (UC10-4F10-11). Antibodies purchased from eBioscience include PE-Cy7 anti-mouse CD4 (GK1.5) and APC anti-mouse/rat Foxp3 staining set (FJK-16s). Functional grade purified hamster anti-mouse CD3ε (145-2C11), Functional grade purified hamster anti-mouse CD28 (37.51), recombinant mouse IL-2 and TNF were obtained from BD Pharmingen. Bay 11-7082 (Cat#: B5556), and Lipopolysaccharides (rough strains) from Salmonella (LPS) (Cat#: L9764) was purchased from Sigma-Aldrich. Sulfasalazine (Cat#: S1576) and SB203580 (Cat#: S1076) was obtained from Selleckchem. LIVE/DEAD Fixable Near-IR Dead Cell Stain Kit (for 633 or 635 nm, L10119) was ordered from Thermo Fisher Scientific.

### Cell Purification and *In Vitro* Cell Culture

Mouse lymphocytes were harvested from spleens, axillary lymph nodes, inguinal lymph nodes, and mesenteric lymph nodes. CD4^+^ T cells were purified from lymphocytes by using CD4 (L3T4) microbeads (Miltenyi Biotec, 130-097-145) and MS column (Miltenyi Biotec). MACS-Purified CD4^+^ cells were labeled with CFSE and cells (5 × 10^4^ cells/well) were cultured in a 96-well plate, then stimulated with IL-2 or IL-2 plus TNF, in the presence or absence of SB203580 (1–25 µM) for 3 days. Proliferation of Tregs was assessed by CFSE dilution assay, and the proportion of Foxp3^+^ cells in CD4^+^ subset and TNFR2 expression on Tregs were analyzed with FACS. In some experiments, FACS-sorted CD4^+^CD25^+^ cells (cells purity: 98%, 5 × 10^4^ cells/well) were stimulated with plate-bound anti-CD3ε Ab (10 µg/mL) and soluble anti-CD28 Ab (2 µg/mL) in the presence of TNF (10 ng/mL) or medium alone, with or without 25 µM SB203580, for 3 days. Expression of Foxp3 and TNFR2 were analyzed by FACS.

### *In Vivo* Administration of LPS and SB203580

C57BL/6J mice were injected intraperitoneally (i.p.) with 200 µg of LPS in 0.2 mL PBS. Some mice were treated with SB203580 (25 mg/kg, i.p.) immediately after LPS treatment. SB203580 were dissolved in a stable solvent system (4% DMSO, 30% PEG 300, 5% Tween 80, and 61% ddH_2_O). After 24 and 72 h, mice were sacrificed. The spleens, lymph nodes at axillary, inguinal, and mesenteric regions, and blood were harvested for FACS analysis.

### Flow Cytometry

After blocking FcR, cells were incubated with appropriately diluted antibodies and finally suspended in FACS buffer for cytometric analysis. Acquisition was performed by BD FACSCanto II and BD FACSAria™ Fusion flow cytometer. Data analysis was conducted by using FlowJo software (Tree Star Inc., Ashland, OR, USA).

### Western Blot

MACS-purified CD4^+^CD25^+^ T cells were stimulated with TNF (100 ng/mL), with or without selected inhibitors [SB203580 (SB), Bay 11-7082 (Bay), Sulfasalazine (Sul)] for 30 min. The cells were homogenized in RIPA buffer containing a cocktail of proteinase and phosphatase inhibitors. Protein samples were separated on a SDS-PAGE gradient gel (4–12% Bis-Tris protein gel; Thermo Fisher Scientific) and transferred to PVDF membranes. The blots were blocked with 5% BSA for 1 h and incubated with phospho-p38 antibody (1:1,000; Cell Signaling Technology) and phospho-NF-κB p65 antibody (1:1,000; Cell Signaling Technology) overnight at 4°C. The blots were then incubated in HRP-conjugated secondary antibody (1:3,000) for 1 h at room temperature, developed in ECL solution (Thermo Fisher Scientific) for 1 min, and exposed by G-Box imager. The blots were then incubated in stripping buffer (Thermo Fisher Scientific) at 37°C for 15 min and reprobing with IκBα antibody (1:1,000; Cell Signaling Technology) or p38 antibody (1:1,000; Cell Signaling Technology) or NF-κB p65 antibody (1:1,000; Cell Signaling Technology) or GAPDH antibody (1:3,000; Cell Signaling Technology).

### Statistical Analysis

Comparisons of two groups of data were analyzed by *t* test using GraphPad Prism 6.0. Comparisons of more than two groups of data were analyzed by one-way ANOVA by using GraphPad Prism 6.0 (GraphPad, San Diego, CA, USA).

## Ethics Statement

This study was carried out in accordance with the recommendations of approved guidelines of Animal Research Ethics Committee, University of Macau. The protocol was approved by the Animal Research Ethics Committee of University of Macau.

## Author Contributions

TH, SL, SC, JY, and XW performed the experiments. TH, ZB, and XC designed the experiments and wrote the manuscript. All authors agree to the submission of the manuscript.

## Conflict of Interest Statement

The authors declare that the research was conducted in the absence of any commercial or financial relationships that could be construed as a potential conflict of interest.
