# Supplementary material for: The p38 MAPK Inhibitor SB203580 Abrogates Tumor Necrosis Factor-Induced Proliferative Expansion of Mouse CD4+Foxp3+ Regulatory T Cells
Source: Front Immunol. 2018 Jul 9;9:1556. doi: 10.3389/fimmu.2018.01556 (PMC6046375; doi:10.3389/fimmu.2018.01556)
Supplement: Supplementary file 1 [file Presentation_1.pdf]

Supplementary Material

The p38 MAPK Inhibitor SB203580 Abrogates TNF-Induced Proliferative Expansion of Mouse $CD4^{+}Foxp3^{+}$ Regulatory T Cells

Tianzhen He[1], Shuoyang Liu[1], Shaokui Chen[1], Jingyi Ye[1], Xueqiang Wu[2], Zhaoxiang Bian[3], and Xin Chen[1*]

1. State Key Laboratory of Quality Research in Chinese Medicine, Institute of Chinese Medical Sciences, University of Macau, Macau SAR 999078, China

2. Department of Oncology, Beijing Aerospace General Hospital, Beijing 100076, China

3. School of Chinese Medicine, Hong Kong Baptist University, Kowloon, Hong Kong SAR, China.

* Corresponding author: Dr. Xin Chen, Institute of Chinese Medical Sciences, University of Macau, Avenida da Universidade, Taipa, Macau SAR 999078, China.Tel.: (853) 8822 4513; Fax: (853) 2884 1358. E-mail address: xchen@umac.mo

1.1 Supplementary Figures

Supplementary Figure 1

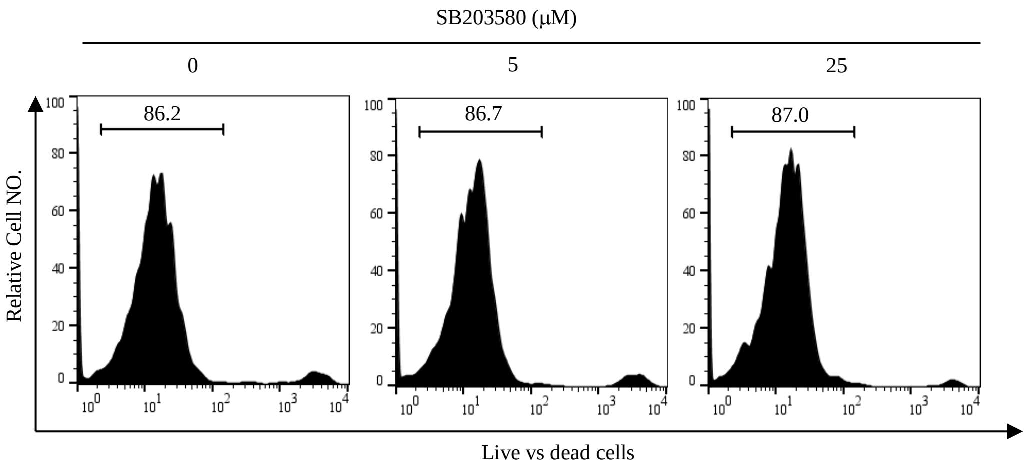

Effect of SB203580 on the viability of cultured CD4$^+$ T cells. CD4$^+$ cells were isolated from LNs and spleen of normal C57BL/6J mice. The cells were labeled with CFSE and cultured in the presence of IL-2 + TNF (10 ng/mL), with different concentrations of SB203580 (0, 5, 25 μM). After 72 hours, cells were collected and stained with LIVE/DEAD dye, and their viability was analyzed by FACS. The left peaks represent live cells. Data shown are representatives of at least three separate experiments with similar results.

Supplementary Figure 2

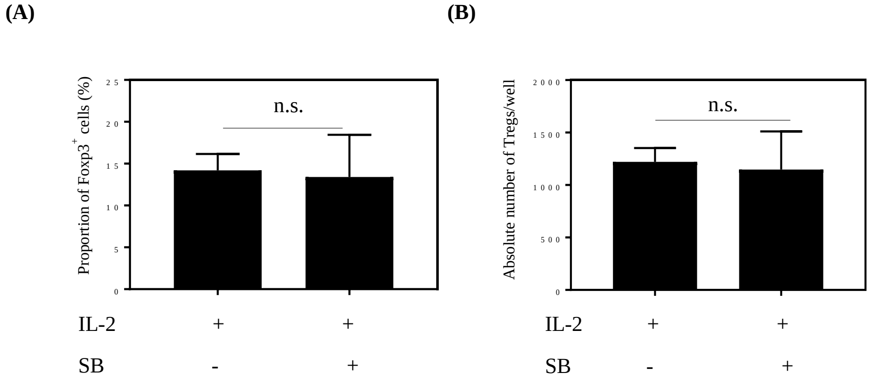

Effect of SB203580 (SB) on the proportion and number of Foxp3$^+$ Tregs in CD4$^+$ cells cultured with IL-2 alone. CD4$^+$ cells were isolated from LNs and spleen of normal C57BL/6J mice. The cells were cultured in the presence of IL-2 (10 ng/mL), with or without 25 μM SB203580. After 72 hours, the proportion of Foxp3$^+$ cells was analyzed by FACS (A). The number of Foxp3-expressing Tregs was calculated (B). Data (N=3, Means ± SEM) shown are representatives of at least three separate experiments with similar results. n.s.: Not significant statistically.

Supplementary Figure 3

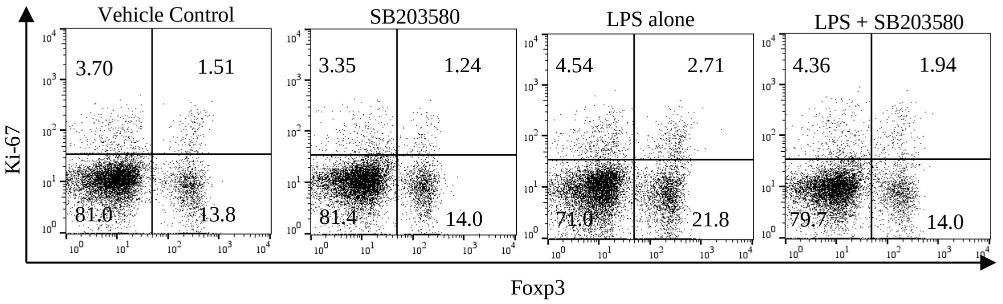

Effect of SB203580 on the expansion of Tregs in mice. Normal C57BL/6J mice were injected with 200 µg of LPS (i.p.) or PBS, and treated with or without SB203580 (25 mg/kg/d, i.p.). The spleen and lymph nodes were harvested 72 hours after treatment. Expression of Ki-67 by Foxp3$^-$ and Foxp3$^+$ cells was analyzed by FACS. Number in the dot plots shows the proportion of cells in the respective quadrants.

Supplementary Figure 4

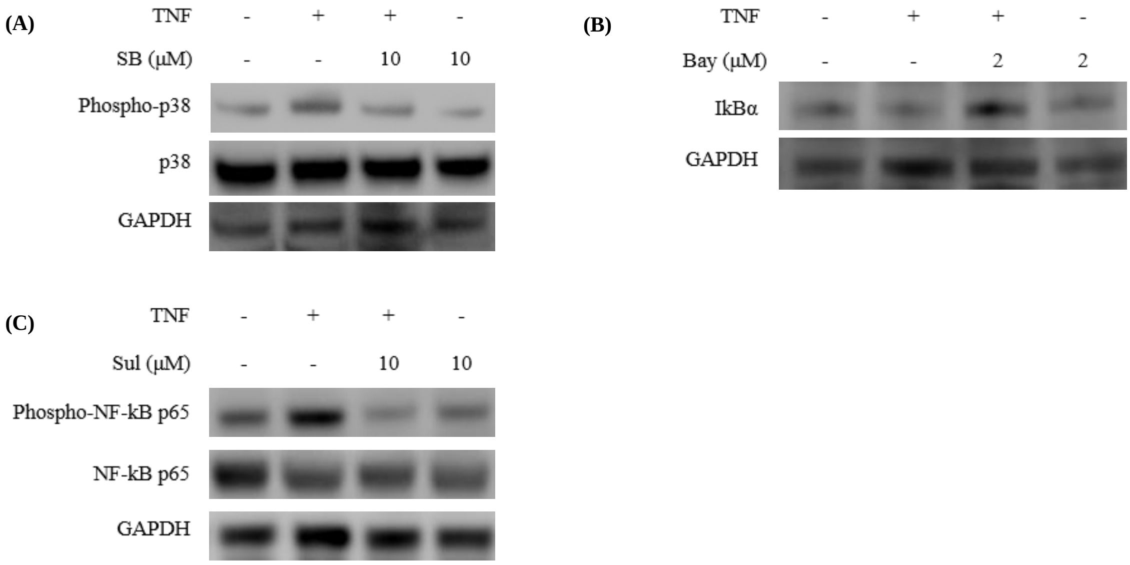

Effects of SB203580, Bay 11-7082 and Sulfasalazine on TNF-induced activation of p38 MAPK and NF-κB pathways. Mouse CD4$^+$CD25$^+$ T cells were stimulated with TNF (100 ng/mL) with or without selected inhibitors (SB203580 (SB), Bay 11-7082 (Bay), Sulfasalazine (Sul)) for 30 min. The activities of p38 and NF-κB pathways were determined using phospho-specific antibodies; protein levels were determined by anti-p38, anti-IκBα and anti- NF-κB p65 antibodies, respectively. Data shown are representative of at least three independent experiments.

1.2 Supplementary Table

Supplementary Table 1.

**Gene Ontology-Biological Processes**	**p-value**	**Gene Symbols**
Activation of MAPK activity	0.00003399	|**MAP2K3(MKK3)**|TAB2|TRAF6|FRS2|MAPKAPK3|MAP3K2|IRAK2|TGFA|**MAPK14(p38α)**|TNF|MAP2K6|TAB3|CHRNA7|DRD4|TLR4|NOD2|NTF3|P2RX7|WNT5A|IRAK1|
Positive regulation of NF-κB activity	0.000002195	|TAB3|PYCARD|CFLAR|TLR2|NFKBIA|NFKBIB|TRAF1|IKBKB|AMH|**NFKB1**|**TRAF2**|IRAK2|**NFKB2**|ICAM1|TRAF6|TNF|FER|TAB2|NOD2|WNT5A|TLR4|IRAK1|ADAM8|PSMA6|

Gene ontology(GO) analysis of MAPK and NF-κB activity in TNF-treated human Tregs. GSE18893, a profile dataset generated by Nagar M, Jacob-Hirsch J and Goldstein I, was downloaded from GEO website (www.ncbi.nlm.nih.gov/geo/query/acc.cgi?acc=GSE18893). Genes significantly differentially expressed (DE) between TNF-treated human Tregs and untreated Tregs was evaluated with Gene Ontology enrichment analysis using web-based online tool GCBI (www.gcbi.com.cn/gclib/html/index). The key genes associated with the activation of p38 MAPK and NF-κB pathway by TNF stimulation are marked in bold.

Supplementary Table 2

**Pathway Name**	**p-value**	**Gene Symbols**
MAPK signaling pathway	0.000000000253	|BRAF|DUSP1|CHP2|HSPA6|RASGRP3|GADD45A|FLNB|FGF22|MAPK8IP2|**MAPK14(p38α)**|PLA2G4C|MAX|FLNC|DUSP5|TAB2|BDNF|SOS2|RELB|MAP3K2|MAP2K6|**MAP2K3(MKK3)**|SOS1|FGF3|TAOK1|ARRB2|MAP3K8|RASGRP1|MECOM|NTF3|MAPKAPK3|RASGRP4|STMN1|RRAS2|PAK2|TNF|FGF19|**TRAF2**|JUN|NGF|PRKACA|IKBKB|TP53|NFKB2|NFKB1|TRAF6|IL1R1|MYC|GADD45B|DUSP7|NFATC2|FAS|
NF-κB signaling pathway	0.000000000000730	|**TRAF2**|ATM|**NFKB2**|**RELB**|TRAF1|TAB2|IL1R1|LCK|CCL13|LTA|TAB3|TLR4|TNFAIP3|DDX58|PARP1|CFLAR|NFKBIA|BCL2L1|LTB|TNFSF13B|GADD45B|IRAK1|IKBKB|CSNK2B|TRAF3|TNF|**NFKB1**|BIRC2|CXCL2|TRAF6|BIRC3|ICAM1|

Pathway analysis of MAPK and NF-κB activity in TNF-treated human Tregs. Analysis of GSE18893 file with Kyoto Encyclopedia of Genes and Genomes (KEGG) pathway enrichment was performed with GCBI. The key genes associated with downstream of p38 MAPK and NF-κB pathway in TNF-treated Tregs are marked in bold.
